# A Hybrid Analytical/Numerical Model for the Characterization of Preferential Flow Path with Non-Darcy Flow

**DOI:** 10.1371/journal.pone.0083536

**Published:** 2013-12-30

**Authors:** Sen Wang, Qihong Feng, Xiaodong Han

**Affiliations:** 1 Department of Petroleum Engineering, China University of Petroleum (East China), Qingdao, China; University of Kansas, United States of America

## Abstract

Due to the long-term fluid-solid interactions in waterflooding, the tremendous variation of oil reservoir formation parameters will lead to the widespread evolution of preferential flow paths, thereby preventing the further enhancement of recovery efficiency because of unstable fingering and premature breakthrough. To improve oil recovery, the characterization of preferential flow paths is essential and imperative. In efforts that have been previously documented, fluid flow characteristics within preferential paths are assumed to obey Darcy's equation. However, the occurrence of non-Darcy flow behavior has been increasingly suggested. To examine this conjecture, the Forchheimer number with the inertial coefficient estimated from different empirical formulas is applied as the criterion. Considering a 10% non-Darcy effect, the fluid flow in a preferential path may do experience non-Darcy behavior. With the objective of characterizing the preferential path with non-Darcy flow, a hybrid analytical/numerical model has been developed to investigate the pressure transient response, which dynamically couples a numerical model describing the non-Darcy effect of a preferential flow path with an analytical reservoir model. The characteristics of the pressure transient behavior and the sensitivities of corresponding parameters have also been discussed. In addition, an interpretation approach for pressure transient testing is also proposed, in which the Gravitational Search Algorithm is employed as a non-linear regression technology to match measured pressure with this hybrid model. Examples of applications from different oilfields are also presented to illustrate this method. This cost-effective approach provides more accurate characterization of a preferential flow path with non-Darcy flow, which will lay a solid foundation for the design and operation of conformance control treatments, as well as several other Enhanced Oil Recovery projects.

## Introduction

During long-term waterflooding, the pore structure of a poorly consolidated sandstone formation will change significantly because of fluid-solid interactions, such as sand production [1] and clay erosion [2]. Therefore, the preferential flow path, which has also been termed the high-permeability streak or thief zone, is commonly formed in reservoirs because sand or clay particles have been eroded by fluid-induced force [3]. Existence of preferential flow paths will lead to inefficient water injection, unstable fingering and premature breakthrough of polymer or steam, all of which will prevent the recovery efficiency from being further enhanced. Accordingly, the identification and characterization of preferential flow paths have become imperative for the Enhanced Oil Recovery process in reservoir development [4].

Approaches that can be applied to characterize the preferential flow path involve interwell surveillance [5]–[8], well logging [9], [10], reservoir engineering method [11]–[14] and well testing [15]–[18]. Darcy's law, which is the basis and principle of almost all of these methods, is always employed to depict fluid flow behavior in the preferential path. However, the influence of non-Darcy effect, especially for the high-rate wells with an ultra-high permeability streak, has been increasingly suggested for fluid flow within this path [18]–[20].

Our objectives in this work can be summarized as follows: (1) to examine whether non-Darcy flow behavior can occur in a preferential path; (2) to provide a hybrid analytical/numerical model to investigate the pressure transient response, which couples the non-Darcy effect of the preferential path with the Darcy flow of the reservoir; and (3) to present an available approach to characterize the preferential path with non-Darcy behavior using a pressure transient test.

## Theoretical Consideration

Accurately describing the fluid flow phenomenon within the preferential path is essential for precise characterization and successful treatment project design. Flow through a preferential path is usually assumed to obey Darcy's equation, i.e., the pressure gradient is linearly proportional to the superficial velocity. Nevertheless, it's increasingly recognized that the non-Darcy effect should not be ignored because a preferential path always has an extremely strong transport capacity [4], [18]–[20]. For example, tests from several poorly consolidated reservoirs in Chinese oilfields indicate that the effective permeability of interwell formations can reach up to 10–150 μm^2^, and tracer advance speed can be as high as 10–100 m/h, which is absolutely incredible for Darcy flow [21], [22]. Therefore, in this section, using the criteria proposed by Zeng and Grigg [23], non-Darcy flow in the preferential path is identified with tracer test data from the Gudong Oilfield.

Darcy's law is widely applied to characterize fluid flow in porous media. However, a large number of experimental observations [24] and numerical simulations [25], [26] have demonstrated that Darcy's law should be restricted to viscous flow conditions or, more precisely, to low values of the Reynolds number (*Re*), which is given by Eq. (1).
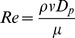
(1)


In this formula, *ρ* is the fluid density, kg/m^3^; *v* is fluid superficial velocity, m/s; *D_p_* is the grain diameter, m; and *μ* is the fluid viscosity, Pa·s.

As the Reynolds number increases, due to the contribution of inertial forces to laminar flow through the void space, the distribution of streamlines along the direction orthogonal to the main flux becomes more homogeneous, which causes flow deviations from Darcy's law [25]. The deviation from the classical linear relationship is defined as non-Darcy flow, which can be quantified macroscopically by an empirical formula named the Forchheimer equation [27],
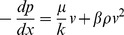
(2)where *p* is the fluid pressure, Pa; *x* is the space coordinate in the fluid flow direction, m; *k* is the permeability of the porous media, m^2^; *β* is an experimentally derived parameter called the non-Darcy flow coefficient or inertial coefficient. Non-Darcy flow behavior has been recognized as a common and important phenomenon in the development of hydrocarbon reservoirs and geothermal resources [28], especially for the fractured wells [29] and the near-well region of high-rate gas wells [30]. The significant influences of nonlinear flow on the well productivity and reservoir performance have been analyzed and confirmed [31], [32].

Inconsistent with the sudden passage from laminar to turbulent flow in conduits and channels, where there is a critical condition separating both regimes, the transition from linear to nonlinear flow behavior is more likely to be gradual for the transport in porous media [25], [33], [34]. To identify the onset of non-Darcy flow, several criteria have been proposed in previous work [23], [25], [35]–[49], which can be summarized in Table 1. Table 1 shows that there are roughly three types of criteria for the determination of inertial flow beginning through porous media: Type 1 represented by the Reynolds number (*Re*), and the critical value varies from 0.1 to 100; Type 2 by the Forchheimer number (*Fo*) ranging from 0.005 to 0.2; Type 3 by various types of modified Reynolds numbers (*MRes*), and the critical value varies with different definitions. Since the Reynolds number has its root from a similar criterion for turbulent flow in pipes, both Type 1 and Type 3 have been employed primarily for packed particles and unconsolidated or poorly consolidated sands, for which a characteristic length is available. Unfortunately, without a well-defined characteristic length, these criteria cannot be employed. However, as recommended by Zeng and Grigg [23], the Forchheimer number (*Fo*), which is defined by
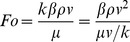
(3)is of explicit definition, rational physical meaning, and general applicability [50]. Eq. (3) illustrates that the Forchheimer number represents the ratio of the pressure gradient consumed by fluid-solid interaction *βρv* ^2^ to that by viscous resistance, *μv/k*. For any types of porous materials, as long as the permeability and non-Darcy coefficient are obtained either by experimental techniques or by empirical models, the Forchheimer number can be available. Thus, with interwell tracer test data from the Gudong Oilfield, the Forchheimer number is employed as the criterion to determine whether non-Darcy flow will occur in a preferential path.

**Table 1:** A brief summary of criteria for non-Darcy flow in porous media.

Parameter	Source	Critical Value	Investigation Method
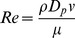	Chilton and Colburn (1931) [35]	40–80	Experiments on packed particles
	Fancher and Lewis (1933) [36]	10–1000 for unconsolidated, 0.4–3 for loosely consolidated	Crude oil, water and air through unconsolidated sands, lead shot, and consolidated sandstones
	Tek (1957) [37]	1.0	Air, water flow through Woodbine, Wilcox, Warren and 3^rd^ Venango sands
	Bear (1972) [38]	3–10	Review and analysis
	Scheidegger (1974) [39]	0.1–75	Review and analysis
	Dybbs and Edwards (1984) [40]	1–10	Experiments in fixed beds of arranged spheres and cylinders
	Blick and Civan (1988) [41]	100	Simulation of capillary-orifice model
	Du Plessis and Masliyah (1988) [42]	3–17	Representative unit cell simulation
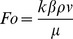	Green and Duwez (1951) [43]	0.1–0.2	N_2_ flow experiments through four different porous metal samples
	Ma and Ruth (1993) [44]	0.005–0.02	Diverging-converging model simulation
	Andrade et al. (1999) [25]	0.01–0.1	Simulation in disordered porous media
	Zeng and Grigg (2006) [23]	0.11	Review and theoretical analysis
	Chukwudozie et al. (2012) [45]	0.02–0.08	Lattice Boltzmann simulation of 3D realistic image-based porous media
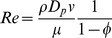	Ergun (1952) [46]	3–10	Gas flow experiments through packed particles
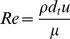	Ma and Ruth (1993) [44]	3–10	Diverging-converging model simulation
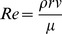	Thauvin and Mohanty (1998) [47]	0.11	Simulation of a pore network model
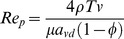	Comiti et al. (2000) [48]	4	Theoretical prediction and experimental data published previously
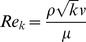	Newman and Yin (2011) [49]	0.1–0.3 for cubic flow, 1–3 for Forchheimer Equation	Lattice Boltzmann simulation of stochastically generated 2D porous media

In this table, *a_vd_* is the dynamic specific surface area, m^−1^; *d_t_* is the throat diameter, m; *r* is the radius of pore throat, m; *T* is the hydraulic tortuosity, dimensionless; *u* is the fluid intrinsic velocity, m/s; *Ф* is the porosity, dimensionless.

**Figure 1:**
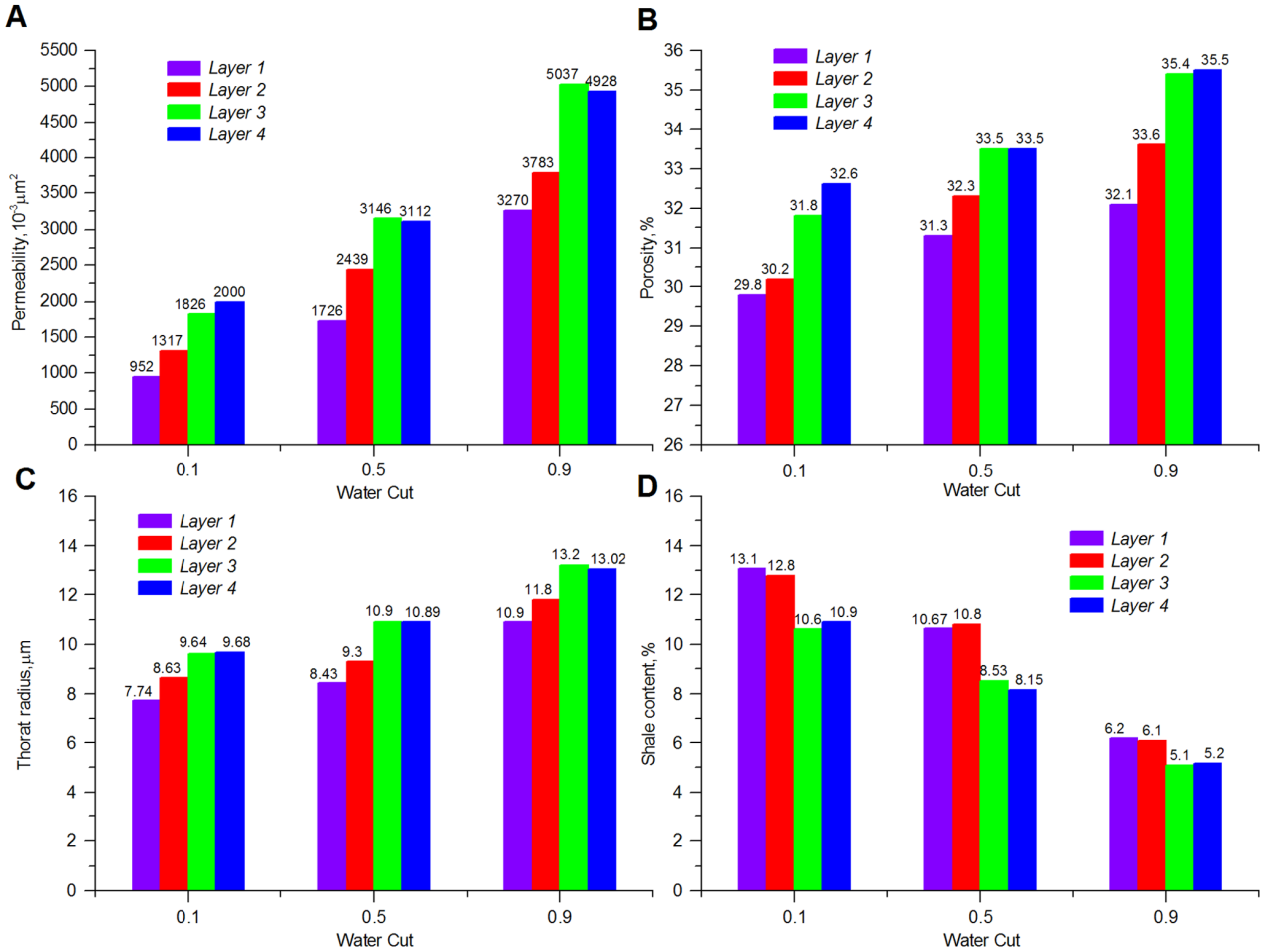
Formation parameter variation for the Gudong Oilfield during long-term waterflooding.

Fig. 1 shows changes of formation parameters from core analysis of the Gudong Oilfield, which has been developed by waterflooding for 26 years. From Fig. 1, it can be observed that along with the increment in the water cut, the permeability, porosity, and pore/throat radius of all the layers are experiencing a dramatic increase, whereas the content of shale is decreasing. Taking the formation permeability of *Layer 4* as an example, at the initial stage (water cut  = 10%), its permeability is only 2000×10^−3^ μm^2^. However, when the water cut increases to 90%, the permeability has become 4928×10^−3^ μm^2^, which is almost 2.5 times as large as the initial value. Analysis of injector/producer production performance also demonstrates that large numbers of preferential flow paths (confirmed by the interwell tracer test) have evolved in this oilfield. Recent statistics from 56 well pairs in this field reveal that in 82% of the tracer tests, the tracer advance speed can reach as much as 15–82.5 m/h, i.e., the tracer can be detected from the production liquid of observation wells several hours after it was injected [22].

To determine whether non-Darcy behavior has emerged in a preferential flow path using the Forchheimer number, estimating the inertial coefficient is essential. Due to the unavailability of experimental data, an empirical model is applied to calculate the non-Darcy coefficient, which assumes a direct correlation of this parameter to fundamental properties of the porous media, such as permeability and porosity. Although tortuosity is also a key factor influencing non-Darcy behavior and is included in some correlation formulas [51], the tortuosity of the preferential flow path is unavailable; therefore, empirical models with tortuosity are not used here.

**Figure 2:**
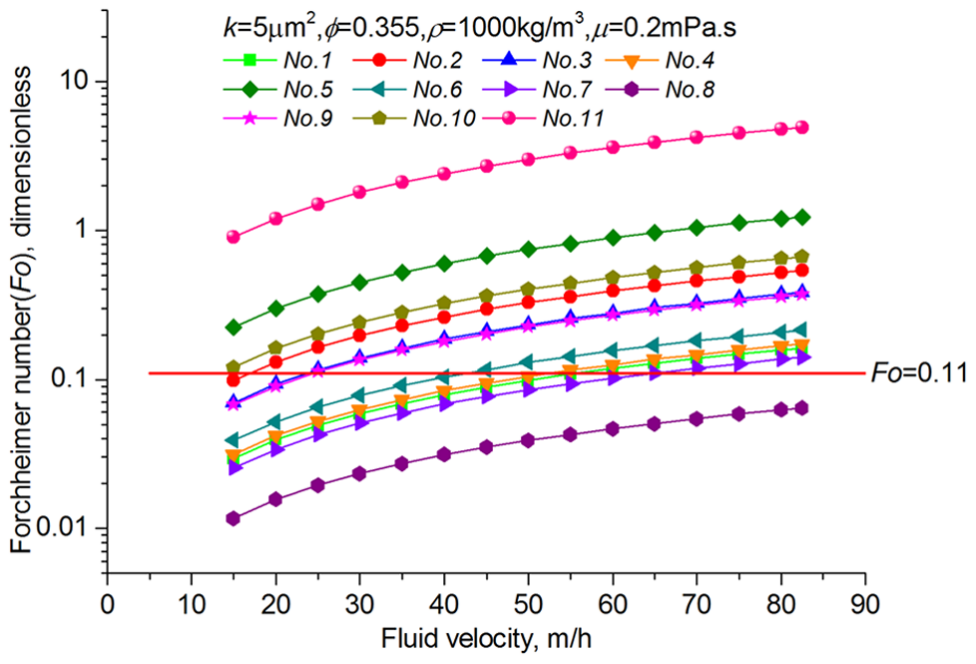
Calculated Forchheimer number for tracer advance speed ranging from 15 m/h to 82.5 m/h and the comparison with the non-Darcy flow criterion.

Table 2 provides a sample of correlations for non-Darcy coefficients from previous studies [24], [46], [52]–[58]. By substituting data from the block of interest (*k* = 5μm^2^, *Ф*  = 35.5%), inertial coefficients are estimated for each model and shown in Table 3. The values of non-Darcy coefficients obtained with different formulas are quite distinct from one another, which makes it difficult to provide definite interpretation. According to the interwell tracer test data from the Gudong Oilfield, with each *β* listed in Table 3, the Forchheimer number is calculated for different tracer advance speeds ranging from 15 m/h to 82.5 m/h (Fig. 2). The red line of critical Forchheimer number 0.11 is also plotted in this figure, corresponding to a 10% non-Darcy effect [23], [59]. Comparison of these plots in Fig. 2 reveals that for almost all of the inertial coefficients estimated with these formulas (except *No. 8* proposed by Coles and Hartman (1998)), fluid flow above a critical velocity (*v_c_*) within a preferential path will experience non-Darcy behavior. Referring to the inertial coefficients in Table 3, larger *β* values are observed to lead to smaller critical velocities. This picture has therefore demonstrated that if the fluid velocity equals or exceeds the critical value, non-Darcy flow will occur in the preferential flow path, consistent with experimental research of Liu et al. [20]. Description technology for the preferential path with Darcy flow can be found in previous studies [5]–[18]. However, no documented effort to take the non-Darcy flow effect into account in the characterization of a preferential flow path has been observed.

**Table 2:** Empirical models for non-Darcy coefficient estimation.

No.	Source	Empirical model	Investigation Method
1	Ergun (1952) [46]	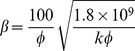	CO_2_, N_2_, CH_4_ and H_2_ flow through various sizes of spheres, sands, and pulverized coke
2	Janicek and Katz (1955) [52]	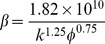	Flow through sandstone, limestone and dolomite
3	Geertsma (1974) [53]	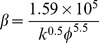	Both consolidated and unconsolidated sandstone, limestone and dolomite
4	MacDonald et al. (1979) [54]	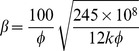	Experimental data from previous work, including spherical glass beads, cylindrical fiber beds and consolidated media
5	Pascal et al. (1980) [55]	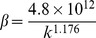	Multirate field test of low permeability hydraulically fractured wells
6	Jones (1987) [24]	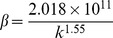	Experiments of vuggy limestone, crystalline limestone and fine-grained sandstone
7	Coles and Hartman (1998) [56]	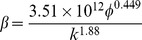	Sandstone and limestone samples for flow testing using the same porosity method
8	Coles and Hartman (1998) [56]	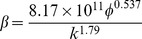	Sandstone and limestone samples for flow testing using the simultaneous equations
9	Li et al. (2001) [57]	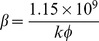	Numerical simulation of N_2_ flowing at various rates through wafer-shaped Berea sandstone
10	Friedel and Voigt (2006) [58]	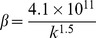	Experimental data from previous studies
11	Friedel and Voigt (2006) [58]	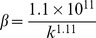	Experimental data from fractures with a variety of proppants

In formulas cited above, *k* appears in 10^−3^μm^2^.

**Table 3:** Non-Darcy coefficient estimated by different empirical formulas.

Formula No.	1	2	3	4	5	6	7	8	9	10	11
Value of *β*, 10^5^m^−1^	2.8367	9.4121	6.6935	3.0211	21.441	3.7284	2.4507	1.1208	6.4789	11.597	86.206

## Methods

### Physical Model and Assumptions

**Figure 3:**
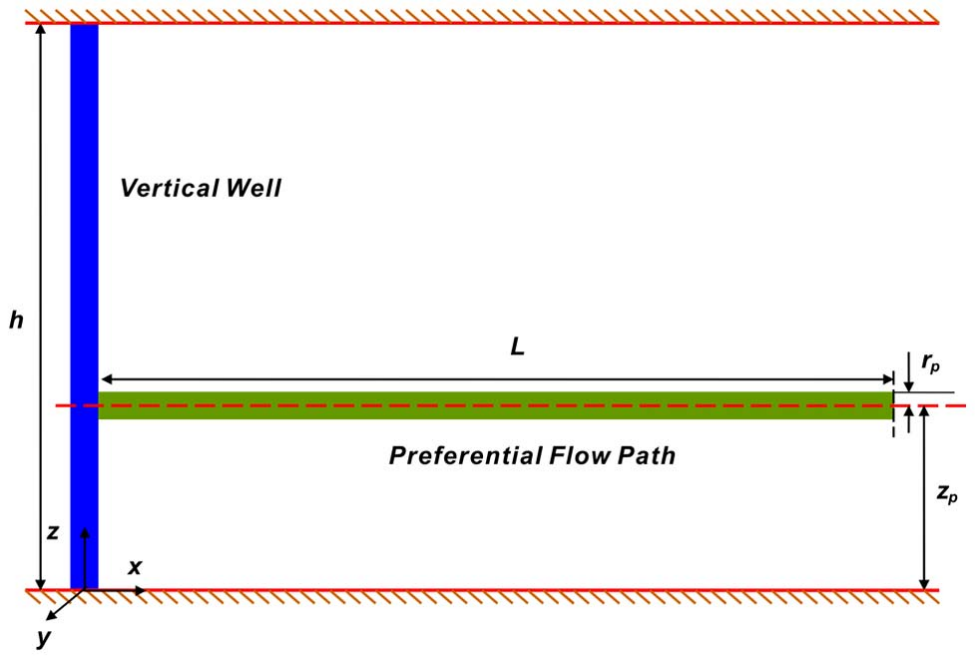
Schematic diagram for a vertical well intersected by a preferential flow path.

Preferential flow path is commonly believed to be a tubular porous medium with extremely high permeability and large pore/throat radius, in which fluid flow is dominant for the whole reservoir [4], [13]. Fig. 3 presents the diagrammatic representation of a vertical well intersected by a preferential flow path. Although the mathematical model proposed in this work is completely general and can be extended to various combinations of boundary conditions, we will concentrate on an infinite slab reservoir with impermeable upper and lower strata.

The compressibility of liquid in the formation can be attributed mainly to the content of gas, i.e., free gas and solution gas [60]. Predetermined by the geological conditions, less dissolved and free gas is contained in the liquid of poorly consolidated or unconsolidated sandstone formations, where the preferential flow path is widely formed. Moreover, the preferential flow path always evolves after long-term waterflooding (approximately 10 to 30 years). At that time, the gas has been almost totally exploited such that the content of gas is very small or approaches zero [61]. Thus the oil can be considered slightly compressible [16], [18], [62]–[65].

Compared with several decades of reservoir development, the pressure transient test period is very short (less than seven days), and the intrinsic topological structure of the reservoir formation hardly experiences tremendous variations. The shear therefore does not change during the test and the influence of the shear on the viscosity can be negligible [66]. Although the fluid viscosity is also affected by other physical parameters, such as temperature and pressure, these parameters can also be considered to be invariable during the short test. Hence, assuming that the fluid is of constant viscosity is reasonable [16], [18], [62]–[66].

To sum up, the following assumptions are made to facilitate this mathematical problem:

1. The reservoir is filled with a single-phase, isothermal, slightly compressible fluid, for which the compressibility and viscosity are assumed to be constant.
2. The formation is of two-dimensional anisotropy, and *k*, *k_v_* stand for the horizontal and vertical permeability, respectively.
3. The bottom (*z* = 0) and top (*z* = *h*) boundaries of the formation are impermeable, and the lateral boundaries do not manifest themselves during the time period of interest.
4. The preferential flow path extends horizontally in the *x* direction and is located at the elevation *z_p_* from the lower boundary of the formation.
5. Due to its small volume, one-dimensional (axial) non-Darcy flow is assumed within the preferential flow path.
6. At the intersection point of the preferential flow path with this well (*x = *0), the production rate *q* is assumed to be constant, whereas at the other end (*x = L*), the flow rate is 0.
7. At different times and different locations, the flow rate from the reservoir to the preferential path is different. Fluid enters the preferential flow path along its axial direction at an unspecified rate per unit length, *q_h_*(*x, t*), and the flow rate at any point *x* within the preferential flow path is *q_d_*(*x, t*), then

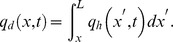



### Dimensionless Variables

Dimensionless variables are defined as follows:

1. Dimensionless pressure drop: 
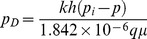
(4)
2. Dimensionless time:
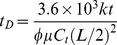
(5)
3. Dimensionless distance:
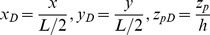
(6)
4. Dimensionless length of the preferential flow path:
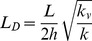
(7)
5. Dimensionless radius of the preferential flow path:
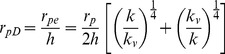
(8)
6. Dimensionless flow rate:
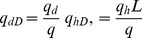
(9)
7. Dimensionless conductivity of the preferential flow path:
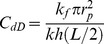
(10)
8. Dimensionless flow rate constant:
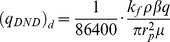
(11)
9. Dimensionless storage coefficient:
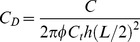
(12)

where *k* and *k_v_* are the horizontal and vertical reservoir permeability, m^2^;*h* is the formation thickness, m; *p_i_* is the initial reservoir pressure, Pa; *q* is the flow rate at the heel of the preferential path, m^3^/d; qh is the influx rate per unit length, m3/(d·m); qd is the cumulative flux at a point in the preferential flow path, m3/d; t is the production time, hours; Ф is the reservoir porosity, dimensionless; *C_t_* is the total compressibility, Pa^−1^;*L* is the length of the preferential flow path, m; *z_p_* is the elevation of the preferential flow path from the lower boundary, m; rp is the radius of the preferential flow path, m; *r_p_* is the quivalent radius of a preferential flow path in an anisotropic reservoir, m; *k_f_* is the permeability of the preferential flow path, m^2^;*C* is the storage constant, m^3^/Pa; *x*, *y*, *z* are the space coordinate in the *x*, *y*, *z*-direction (m), respectively.

### Mathematical Model

**Mathematical model of non-Darcy flow in preferential path:** Fluid flow in a preferential path with non-Darcy behavior can be represented by the Forchheimer equation:
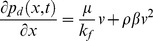
(13)where *p_d_*(*x, t*) is the pressure in the preferential flow path at point *x* at time *t*, Pa.

Fluid velocity in the preferential flow path at point *x* at time *t* is
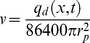
(14)


Substituting Eq. (14) in Eq. (13), we have
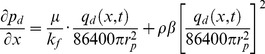
(15)
Eq. (15) can be simplified by applying those aforementioned dimensionless variables
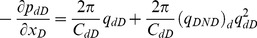
(16)
Eq. (16), which describes the pressure drop per unit length along the preferential flow path, must be integrated to determine the dimensionless pressure at point xD. Integrating Eq. (16) for xD from 0 to xDj yields
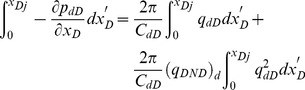
(17)
Eq. (17) can be written as
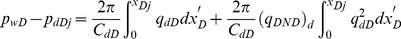
(18)where pw is the pressure at the heel of preferential flow path, Pa.

**Figure 4:**
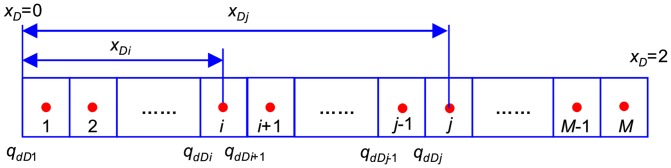
Diagram showing segments with cumulative flux distribution in the preferential flow path.

Eq. (18) is an integral equation, which is very difficult to evaluate directly. Nevertheless, it can be calculated by discretizing the integral in space [67], [68]. The preferential flow path is therefore divided into *M* equal segments (Fig. 4). The flux density is assumed to be constant in each segment, whereas the cumulative flow rate, *q_dD_*, varies from point to point along the preferential path, which can be approximated at point *x*'*_D_* as follows.
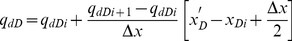
(19)


In this equation, Δ*x*  = 2/*M* is the size of each segment; *i* refers to segment *i*.

From Eq. (18), the integrals can be evaluated separately,
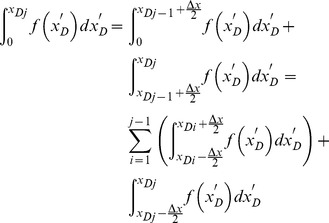
(20)


The following integrals of the polynomials can be calculated
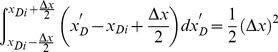
(21)

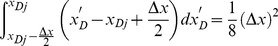
(22)

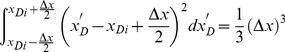
(23)

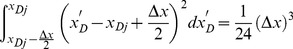
(24)


By applying Eq. (19), one then obtains
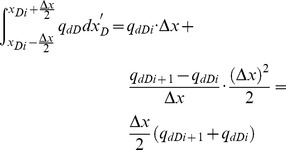
(25)

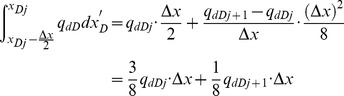
(26)

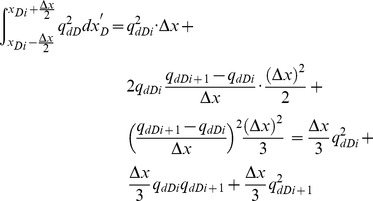
(27)

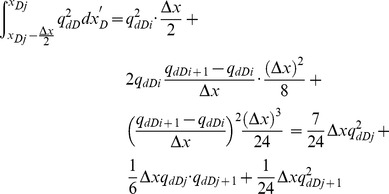
(28)


According to Eq. (20), the integrals in Eq. (18) can be expressed as
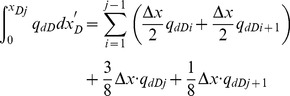
(29)

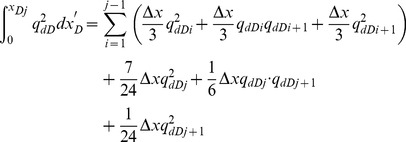
(30)


Now the differential equation of non-Darcy flow in the preferential flow path has been established by Eqs. (18), (29) and (30).

**Mathematical model of Darcy flow in the reservoir:** If the preferential flow path is considered to be a line source, then the dimensionless pressure drop *p_dD_* at point *x_D_* on its surface can be obtained analytically with Green's function [69], [70].
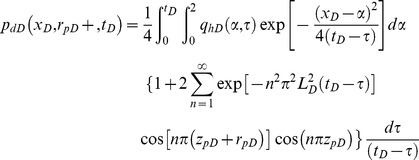
(31)


The dimensionless pressure can be discretized on the surface of the preferential flow path using the same grid and time step as the non-Darcy flow equation to yield the following expression. 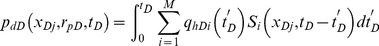
(32)where



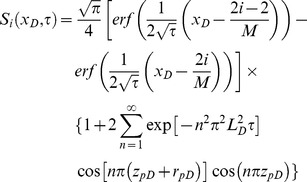
(33)In this equation, *erf* is the error function. Eq. (32) can also be discretized in time as follows.
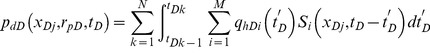
(34)


Here, *N* is the number of time step. If *q_hDi_* is assumed to be constant during the time interval (*t_Dk_* – *t_Dk−_*_1_), then Eq. (34) can be written as
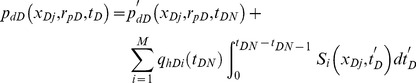
(35)where



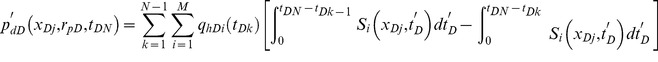
(36)
**Coupling Relationship:** By the continuity of pressure and flow rate on the surface of the preferential flow path, the *p_dDj_* terms in Eqs. (18) and (35) are the same. The mathematical relationship between cumulative flow rate *q_dD_* and flux density *q_hD_* flowing from the reservoir to the preferential path at point *x* can be given by
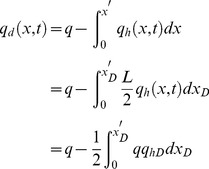
(37)
Eq. (37) can be expressed in dimensionless form as



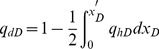
(38)Differentiating Eq. (38) yields
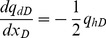
(39)


From the grid we used to discretize the preferential flow path, Eq. (39) can be approximated by
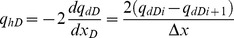
(40)


Thus, substituting Eq. (40) into Eq. (35) yields
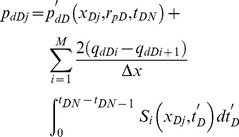
(41)


### Solution Procedure

Substituting Eq. (41) for the term *p_dDj_* in Eq. (18), the following discretized form of non-Darcy flow equation within the preferential flow path is obtained, 
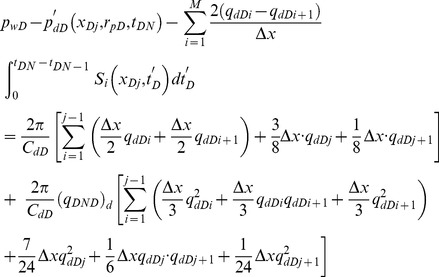
(42)
Eq. (42) represents the dimensionless pressure loss between the vertical well intersection and point *x_Dj_*. Evaluation of Eq. (42) at the center of each segment *x_Dj_* brings about a set of *M* equations with *M*+2 unknown variables: *p_wD_*, *q_dDj_* (*i* = 1,2,*…,M*+1). Two more boundary conditions are therefore required. The first boundary condition can be given by the assumption that the flow rate at the tip far from this well is 0.



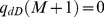
(43)Since the flow rate at the intersection point is equal to *q*, the second equation can be expressed as
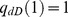
(44)
Eq. (42) can be solved in a forward manner in time because the evaluation of the *p*'*_dD_* term in Eq. (42) needs the knowledge of *q_dDj_* (*i* = 1,2,*…,M*+1) at old time steps *t_D_*_1_,*t_D_*_2_,…,*t_DN−_*_1_. In addition, the set of equations is nonlinear, so the Newton-Raphson iterative scheme is employed at each time step.

Moreover, the following equation can be used to incorporate the effect of storage *C_D_* [71]

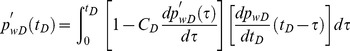
(45)where *p_w_* is wellbore pressure without the storage effect, which is obtained from our model, Pa. The parameter *p*'*_w_* is wellbore pressure with the storage effect at the heel of the preferential flow path, Pa.

Discretizing Eq. (45) yields the following formula [72]

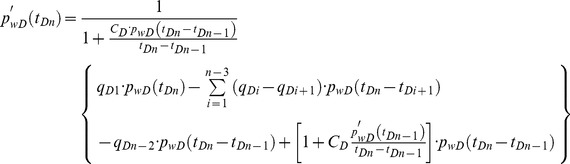
(46)where
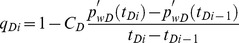
(47)


### Model Verification

**Figure 5:**
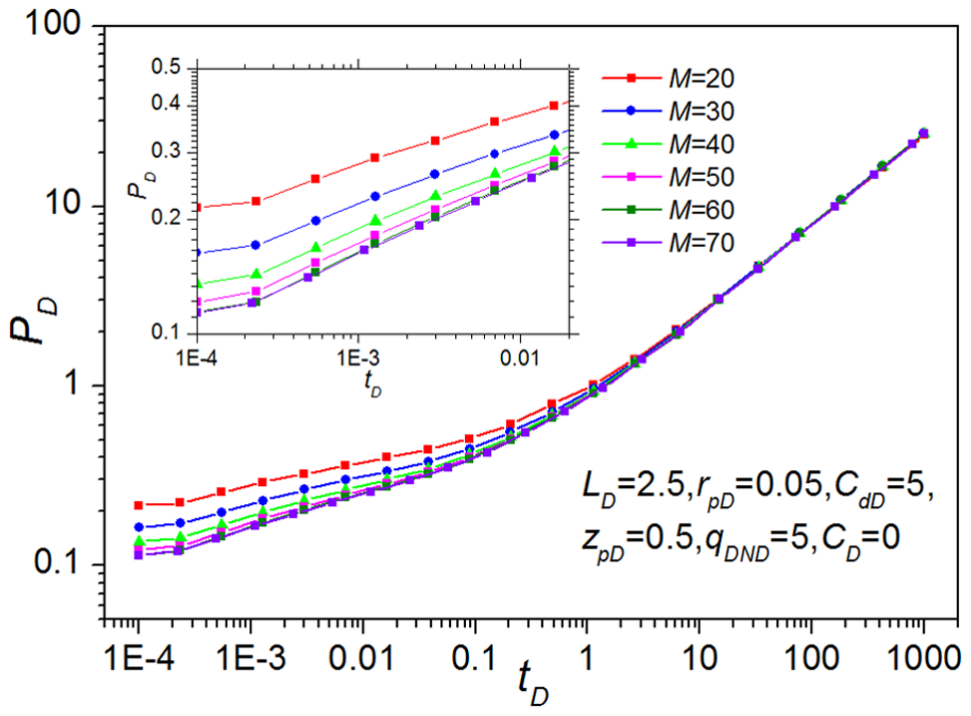
Influence of discrete element numbers on the dimensionless pressure.

For the mathematical model, the appropriate choice of the segment number used to discretize the preferential flow path in space is essential to guarantee the accuracy and efficiency of the fluid flow simulation. Increasing the discrete element number *M* would improve the accuracy, whereas the size of the coefficient matrix needing to be solved also increases dramatically at each time step, leading to a long computation time. To provide a measure, we compare the pressure transient response of different segment numbers for the base-case model. Fig. 5 presents the effect of discrete element numbers on the dimensionless wellbore pressure. The result shows that if the number of segments is greater than or equal to 60, these pressure curves would completely coincide; however, if *M* is less than 60, there is a great difference from the other cases. Therefore, to ensure the accuracy and to reduce computational costs, a value of 60 segments is applied and recommended for the simulation.

For the reason that no transient-pressure solution for a preferential flow path with non-Darcy behavior is available in previous literature, the model presented in this work is verified by examining the analogous case of a horizontal well, which couples wellbore hydraulic and reservoir fluid flow [70]. Because flux distribution dictates both the flow behavior and the pressure performance, we will compare the flux distribution along the preferential flow path with the flux distribution of a horizontal well. Since the preferential path has an extremely high flow capacity, the physical model of the horizontal well is similar to the preferential flow path, except that fluid flowing through the wellbore is pipe flow instead of non-Darcy flow. From this perspective, characteristics of the influx profile should be similar for both the preferential flow path and the finite-conductivity horizontal well.

**Figure 6:**
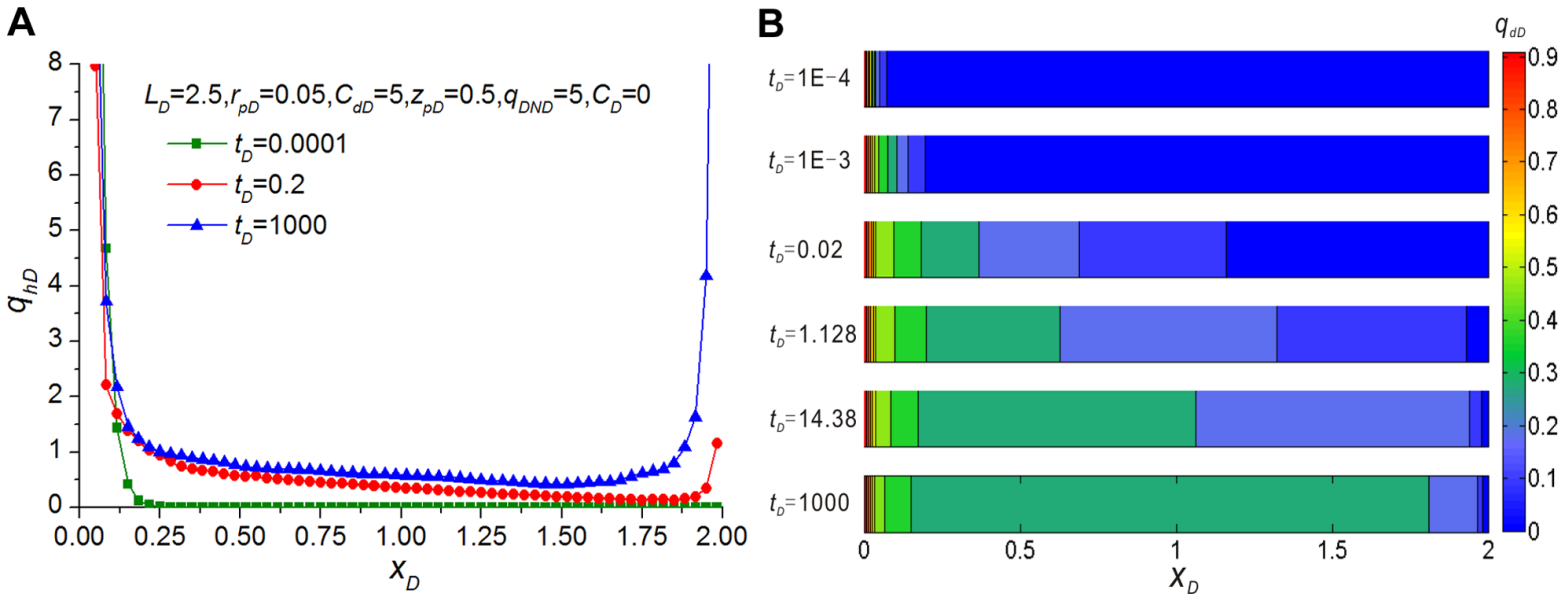
Dimensionless influx rate distribution at *t_D_*  = 0.0001, 0.2 and 1000 (A) and dimensionless flow rate distribution at *t_D_*  = 10^−4^, 10^−3^, 0.02, 1.128, 14.38 and 1000 (B) along the preferential flow path (*L_D_*  = 2.5, *r_pD_*  = 0.05, *C_dD_*  = 5, *z_pD_*  = 0.5, *q_DND_*  = 5, *C_D_*  = 0).

Fig. 6A, showing the dimensionless influx distribution along the preferential flow path at different times, indicates that at early times (*t_D_* ≤ 10^−4^), most of the fluid enters the preferential flow path from the near vicinity of the wellbore, and flow across the tip far from the wellbore intersection is negligible. This observation is in agreement with theoretical analysis because pressure drop always starts from the near wellbore and transmits outwards, which can also be substantiated by the dimensionless flow rate distribution at different times (Fig. 6B). With the time increasing, formation far from the intersection begins to supply fluid gradually, so flux increases in most parts of the preferential flow path except the near intersection. Moreover, the largest increase of flux happens at the tip far from the vicinity of the wellbore. Fig. 6A also reveals that the flux distribution stabilizes at late times (*t_D_* ≥10^3^). At that time, the production rate comes mainly from the vicinity of the formation of the tips because of their larger drainage area. In addition, although the flux profile is a U-shaped curve, it's asymmetric with respect to the mid-point of the preferential flow path, which can be attributed to the influence of non-Darcy flow. Like the frictional pressure drop along a finite-conductivity horizontal well, inertial flow leads to a greater pressure drop at the near vicinity of the wellbore intersection, from which most of the fluid enters the preferential flow path. Therefore, we have attained good qualitative agreement with the influx profile of the finite-conductivity horizontal well, which was proposed by Ozkan et al. [70].

## Results and Discussion

In order to discuss the pressure transient response of a preferential flow path with non-Darcy behavior, we present the results obtained by the hybrid analytical/numerical model developed in this work. The influence of the following parameters on the pressure performance of the preferential flow path is also discussed: length (*L_D_*), radius (*r_pD_*), conductivity (*C_dD_*), vertical location (*z_pD_*), flow rate constant ((*q_DND_*)*_d_*) and storage coefficient (*C_D_*). Then a computer-aided automatic matching algorithm is applied to characterize the preferential flow path using pressure transient analysis.

### Pressure Transient Behavior

**Figure 7:**
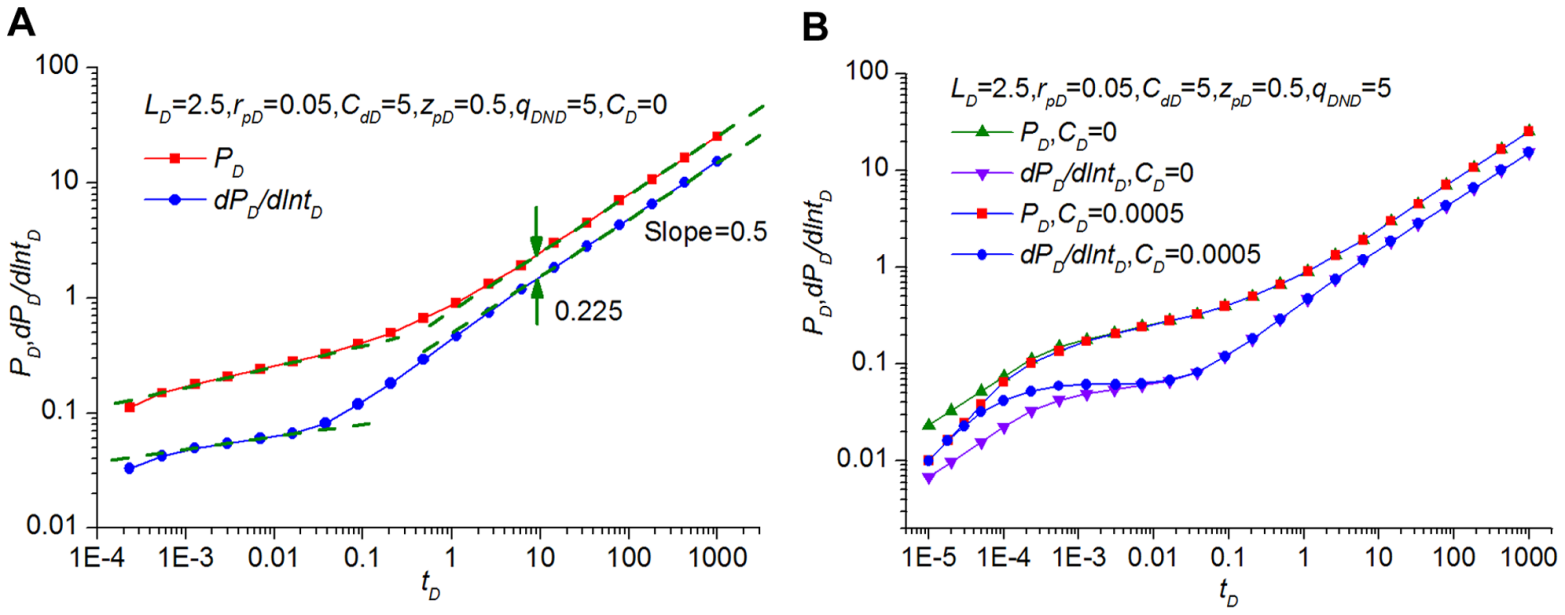
Pressure transient response without (A) and with (B) storage effect.

Fig. 7A shows the dimensionless pressure and derivative responses as a function of time for the preferential flow path without the storage effect. It can be observed that the general pressure transient behavior of this model can be divided into several flow periods.

Stage 1: One-dimensional non-Darcy flow in a preferential path, which is analogous to early-time linear flow in a high-conductivity fractured well. However, because the preferential flow path, which evolves from long-term waterflooding, is always of extremely high conductivity, the flow is so rapid that the linear flow occurs too early to be of practical interest and is therefore not displayed in Fig. 7A. In practice, even a small storage coefficient (*C_D_*  = 5×10^−4^) may mask the characteristics of the early-time one-dimensional flow and the transition regimes, as indicated in Fig. 7B.

Stage 2: At intermediate times, there is a vertical radial flow in the formation and one-dimensional non-Darcy flow within the preferential flow path. This is characterized by an approximately straight line and similar to the radial-linear flow in the horizontal well intercepted by a finite-conductivity vertical fracture (indicated by almost constant derivative response) [73] and the bilinear flow in the vertical well with a finite-conductivity vertical fracture (indicated by a 1/4-slope straight line) [74].

Stage 3: As time increases, a formation linear flow will be observed, which is indicated by a half-slope straight line for both the dimensionless pressure and derivative curves. This flow regime is akin to the linear flow in finite-conductivity vertical fracture communicating with a vertical well over its entire height. However, the gap between pressure *P_D_* and derivative *dP_D_/dlnt_D_* is not *lg*2  = 0.3010 but approximately 0.225. Like the pressure response of the horizontal well, if the ratio of the preferential flow path length to the formation thickness is very large, this regime will last a very long time [75].

Stage 4: At late times, the system reaches a pseudo-radial flow period, and the pressure derivative curve becomes a horizontal line. However, the formation linear flow (Stage 3) always continues for a notably long time; therefore, well testing data for this flow regime are more likely to be unavailable in the oilfield application and not displayed in Fig. 7A.

**Figure 8:**
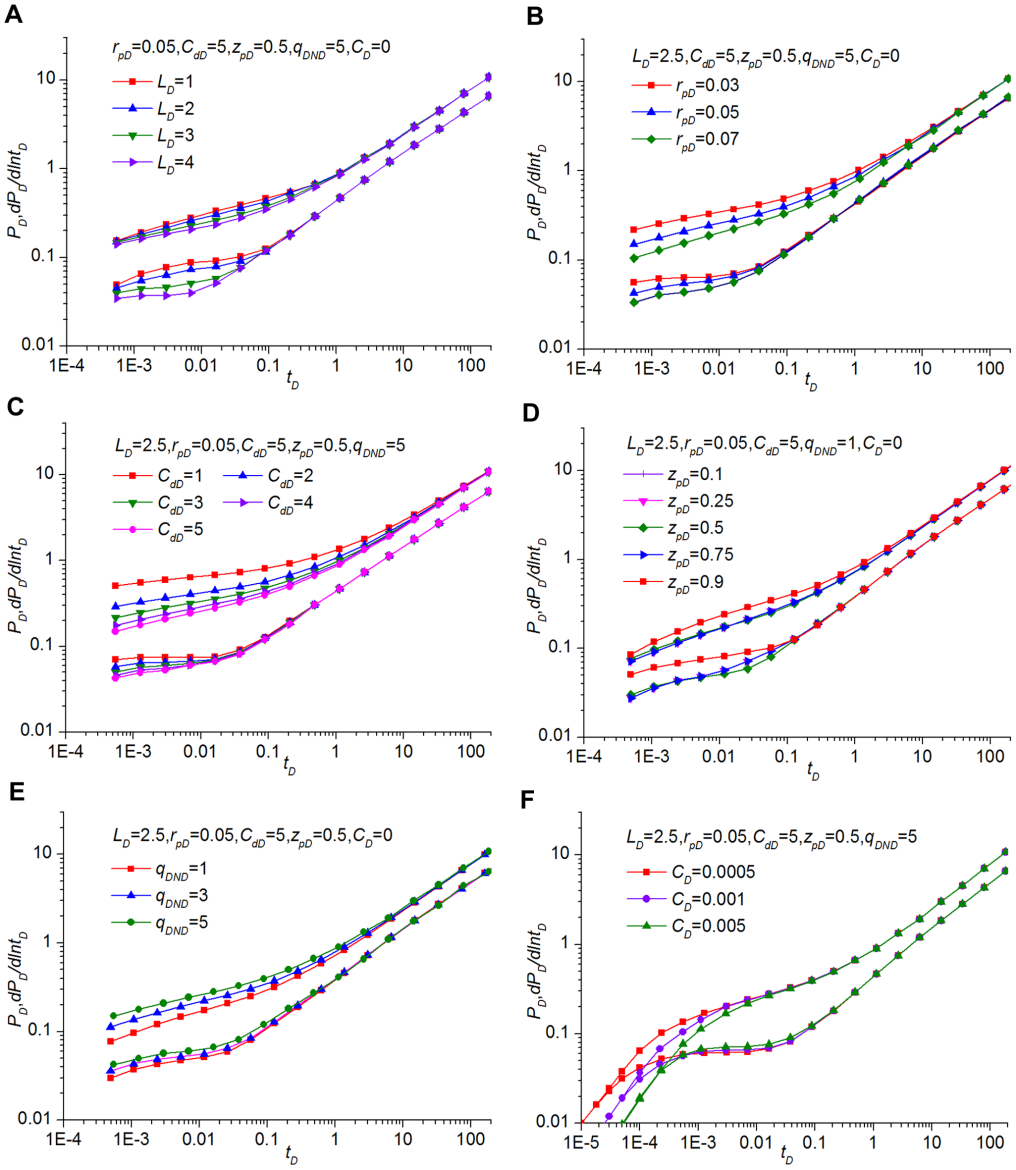
Influence of corresponding parameters on the pressure and derivative. (A) preferential flow path length *L_D_*, (B) equivalent radius *r_pD_*, (C) conductivity *C_dD_*, (D) vertical elevation *z_pD_*,, (E) flow rate constant *q_DND_* and (F) storage effect *C_D_*.

The influence of *L_D_* on the pressure transient behavior is shown in Fig. 8A. The solutions exhibited in this figure are for a preferential flow path with a constant dimensionless conductivity of 5 located mid-height in the reservoir (*z_wD_*  = 0.5), and *k*  =  *k_v_* is also assumed. It indicates that *L_D_* has an effect only upon the early stage, and long-time pressure response is unaffected. Fig. 8A presents that with the decrease of dimensionless length *LD*, the inertial effect of non-Darcy behavior will cause flow choking and increase pressure drop and derivative within the preferential flow path.

The results shown in Fig. 8B indicate that the equivalent radius of the preferential flow path *r_pD_* has a significant effect on the pressure transient performance in the early stage. As the dimensionless equivalent radius decreases, the choking effect will lead to an increasing pressure drop for a given value of *C_dD_*; the smaller the radius, the higher the *P_D_*. For the derivative, as *r_pD_* increases, the pressure drop attributable to the choking flow is insignificant compared with the reservoir, indicating a lower value of the dimensionless derivative.

The pressure response shown in Fig. 8C displays the influence of dimensionless conductivity. Because the permeability, length and radius all have an effect on the conductivity of the preferential flow path, the length *L_D_* and radius *r_pD_* are kept constant. The characteristics of transient-pressure behavior for the preferential flow path with different conductivities are similar. The effect of conductivity concentrates mainly on the early times, yet all the pressure responses coincide at late times. A smaller conductivity will contribute to a larger dimensionless pressure and derivative when *t_D_* ≤1.

Fig. 8D reveals the influence of the dimensionless flow rate constant, which represents the non-Darcy effect on the characteristics of the pressure transient response. The preferential paths with distinct flow rate constants show a similar pressure performance, except that the pressure and derivative curves are slightly different when *t_D_* ≤1. As time decreases, the difference between these plots increases, which can be attributed to the inertial pressure drop caused by non-Darcy flow behavior. The comparison with Fig. 8C illustrates that the effect of *q_DND_* is to lower the conductivity of the preferential flow path, which is consistent with previous study [70]. The pressure and derivative difference for different values of *q_DND_* is detectable at early times because the preferential flow path with larger *q_DND_* behaves as if it has a lower conductivity.

Fig. 8E shows the influence of the vertical location on the pressure performance of the preferential flow path. The vertical location, represented by the dimensionless distance from the lower boundary to the horizontal surface, has a primary effect mainly at the early times, whereas the pressure responses at late times (formation linear flow) are insensitive to the location. When the vertical location is at mid-height in the formation (*zpD*  = 0.5), the dimensionless pressure and derivative are smaller. When the vertical location is at the bottom (*z_pD_*  = 0) or top (*z_pD_*  = 1) of the formation, the pressure and derivative reach the maximum. However, if the vertical location is at 1/4 or 3/4 of the height of the formation (*z_pD_*  = 0.25, 0.75), the dimensionless pressure and derivative are minimum when *t_D_* <0.005. In addition, these preferential flow paths located symmetrically in the vertical direction have identical pressure transient behavior, such as *z_pD_*  = 0.25 and 0.75, *z_pD_*  = 0.1 and 0.9.

Fig. 8F provides information on the influence of the storage effect. The storage affects only the early-time flow period. As *C_D_* increases, the dimensionless pressure decreases, and the pure storage period is lengthened. For the derivative curve, *C_D_* has a great influence on the transition period followed by the formation linear flow. If the storage is high, the dimensionless pressure derivative will increase and display almost flat lines for 0.002 ≤ *t_D_* ≤0.02.

### Characterization of the Preferential Flow Path

For the purpose of dealing with the preferential flow path by successful project design and implementation of conformance control treatment, the prerequisites for further Enhanced Oil Recovery measures, the characterization of the preferential flow path is essential and imperative. As mentioned in the introduction, to the knowledge of the authors, non-Darcy flow behavior is seldom taken into account in the existing works for the description of the preferential path. As shown in Fig. 8 (A–F), all of these type curves have similar shapes; therefore, unique solutions are difficult to obtain manually. To bridge this gap, an automatic matching method has been employed to estimate parameters of the preferential flow path because the computer-aided algorithm offers numerous advantages over the conventional graphical techniques. The automatic history matching technology has such merits as the capability to analyze multi-rate tests and actual data in the transition regions, to avoid inconsistent interpretations and to provide confidence estimates [76]. We use the Gravitational Search Algorithm (GSA) [77] to fit the model and the data as closely as possible by minimizing the sum of the squares of the differences between the measured and model pressures, which is given by
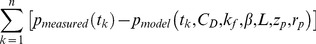
(48)


Conventional gradient-based methods are not efficient and practical for solving optimization problems with a high-dimensional search space because the feasible domain increases exponentially with the problem size [77]. If the objective function is multimodal and/or non-smooth, these methods may readily be trapped into local optima [78]. To overcome these drawbacks, various heuristic approaches, which mimic physical or biological processes in natural phenomena, have been proposed and widely adopted in many different areas [79]–[83]. Among these computational intelligence-based techniques, four well-known global optimization approaches (Genetic Algorithm (GA) [84], Simulated Annealing (SA) [85], Ant Colony Optimization (ACO) [86], and Particle Swarm Optimization (PSO) [87]) have attracted the most attention and application for their novelty and strong searching capacity [88]. However, because of their premature convergence and local stagnation, which are frequently observed in many applications, these algorithms can achieve a better solution for some specific problems rather than others. Searching for novel and efficient optimization methods has therefore been an open problem [77].

Inspired by Newton's law of universal gravitation and mass interaction, a new heuristic optimization approach, Gravitational Search Algorithm (GSA), was proposed by Rashedi et al. in 2009 [77]. According to the theory of Newton, each particle in the universe attracts every other particle with gravitational force directly proportional to the product of their masses [89]. In this algorithm, a set of agents, also called masses, are introduced and considered as objects to find the optimum. The position of each agent denotes a possible solution of the problem, with its mass characterizing the fitness value. All of these agents cooperate with each other using a direct form of communication through the gravitational force, which results in a global movement towards other objects with heavier masses. Heavier agents, which correspond to better solutions, move more slowly than the lighter ones. Ultimately, masses are attracted by the heaviest agents, which provide the optimum solution in the search space [88].

With a flexible and well-balanced mechanism to enhance both exploration (the ability to expand the search space) and exploitation (the ability to find the optimum in the vicinity of a good solution) abilities, this approach is easy to implement and computationally efficient. The high quality performance has been certified to solve a wide range of practical optimization problems, such as filter modeling [88], future oil demand prediction [90], parameters identification [91] and power flow optimization [92]. The capacity of Gravitational Search Algorithm has been evaluated and compared with the Real Genetic Algorithm (RGA), Particle Swarm Optimization (PSO), and Central Force Optimization (CFO) for 23 standard benchmark functions, including unimodal high-dimensional, multimodal high-dimensional, and multimodal low-dimensional types. The results show that in most cases, GSA provides superior results and in all cases, results from GSA are comparable to RGA, PSO and CFO [77]. The same conclusion has also been obtained by other researchers [93]–[94]. Therefore, GSA is employed to solve the optimization problem and to estimate parameters of the preferential flow path. The computational steps of this algorithm can be found in [18], [77], [78], [88], [91]–[94].

### Oilfield Applications

To demonstrate the validity of the approach described in the previous sections, two examples from different oilfields are presented.

**Figure 9:**
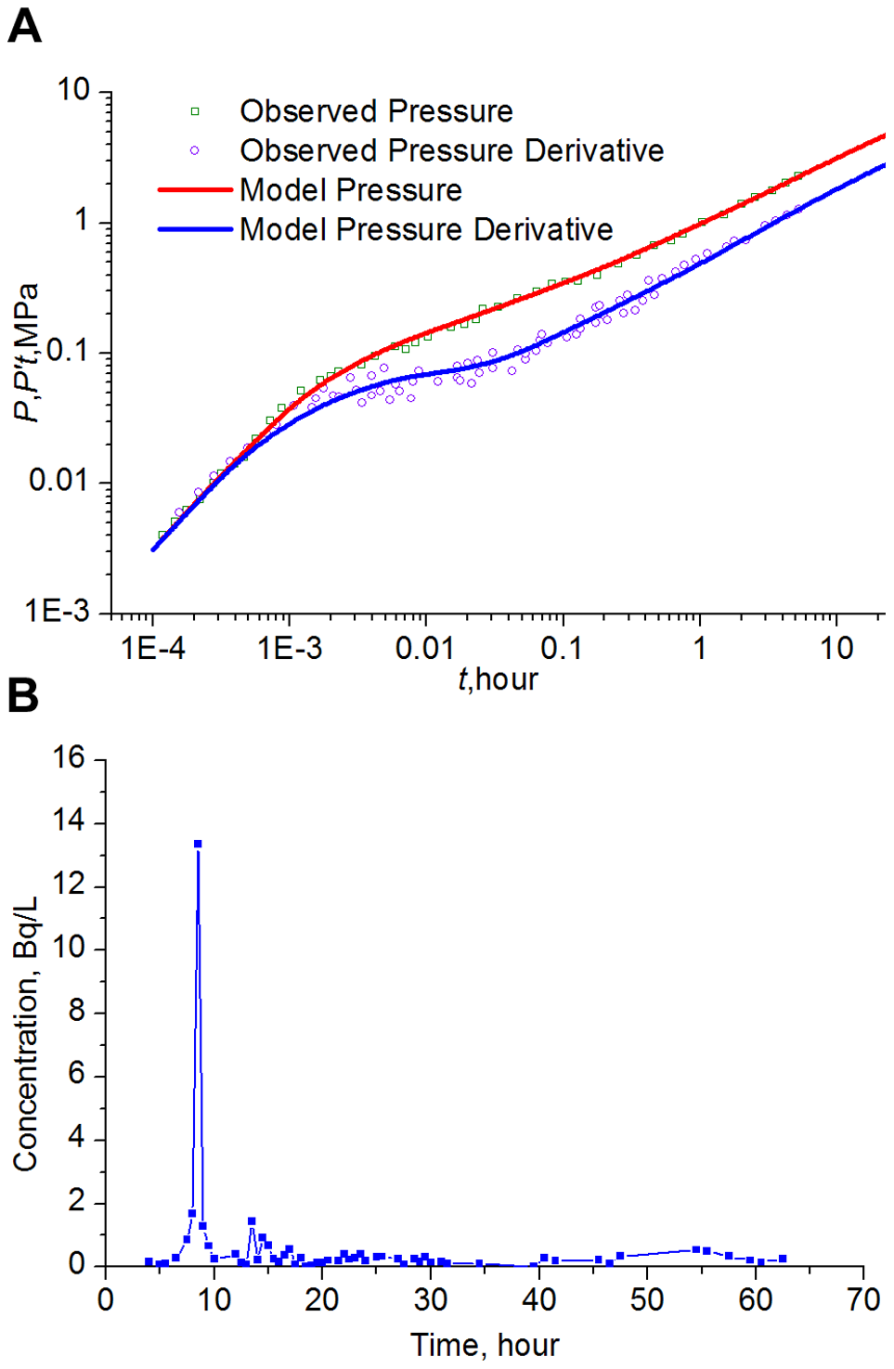
History match of the pressure transient test (A) and interewell tracer concentration curve (B) for Well A1.

**Field Case 1:** Shengli Oilfield, the third largest reservoir in China, is located at the Yellow River Delta in Shandong Province. This poorly consolidated sandstone reservoir has been exploited for more than 30 years. At present, the field water cut is 96.7%, and 34.5% of the oil reserves have been extracted. However, the production performance of many injectors/producers and interwell tracer tests reveal that a few preferential flow paths may have evolved in the formation. Table 4 lists the formation and fluid parameters for Well A1. The observed pressure and derivative are plotted in log-log coordinates in Fig. 9A. Using the automatic history matching technique of the Gravitational Search Algorithm, the computer can obtain the best fit match to the observed data with the presented model. Fig. 9A shows an acceptable agreement has been discovered, and the interpretation parameters of the preferential flow path are given in Table 5. The estimated permeability of the preferential flow path is approximately 89.42 μm^2^ and of extremely high conductivity, so that the strong heterogeneity leads to an incredibly quick premature breakthrough of chemical agents, as observed in the oilfield.

**Table 4:** Formation and fluid parameters for Well A1.

Parameter Name	Symbol	Value	Unit
Flow rate	*q*	111.84	m^3^/d
Fluid density	*ρ*	850	kg/m^3^
Fluid viscosity	*μ*	0.5	mPa·s
Reservoir porosity	*Ф*	0.2514	dimensionless
Reservoir permeability	*k*	0.2487	μm^2^
Formation thickness	*h*	18.5	m
Compressibility	*C_t_*	0.001229	1/MPa

**Table 5:** Parameters of the preferential flow path for Well A1.

Parameter Name	Symbol	Value	Unit
Length of the preferential flow path	*L*	46.85	m
Permeability of the preferential flow path	*k_f_*	89.42	μm^2^
Vertical elevation of the preferential flow path	*z_p_*	7.733	m
Equivalent radius of the preferential flow path	*r_p_*	0.7104	m
Non-Darcy flow coefficient	*β*	5.72×10^6^	m^−1^
Storage constant	*C*	0.024	m^3^/MPa

Polyacrylamide was first injected into the neighbor Well A2, 300 m from Well A1, to block the high-permeability streak and to modify the injection profile. However, only 8.6 hours later, polyacrylamide was observed in the production liquid of Well A1. With the objective of better understanding the interwell connectivity, ^59^Fe tracer was injected from the Well A2. Only 7.5 hours later, the ^59^Fe reached Well A1, and the concentration of ^59^Fe began to increase significantly. Fig. 9B shows the tracer concentration curve for Well A1, and the tracer advance velocity is about 40 m/h, which is very high for this conventional reservoir. Moreover, integration analysis of geological information, Production Logging Test (PLT) and interwell dynamic connectivity from the Capacitance-Resistance Model (CRM) [12] is also consistent with the characterization. The treatment project design based on this interpretation result also shows very good performance, which has demonstrated the validity of this approach.

**Figure 10:**
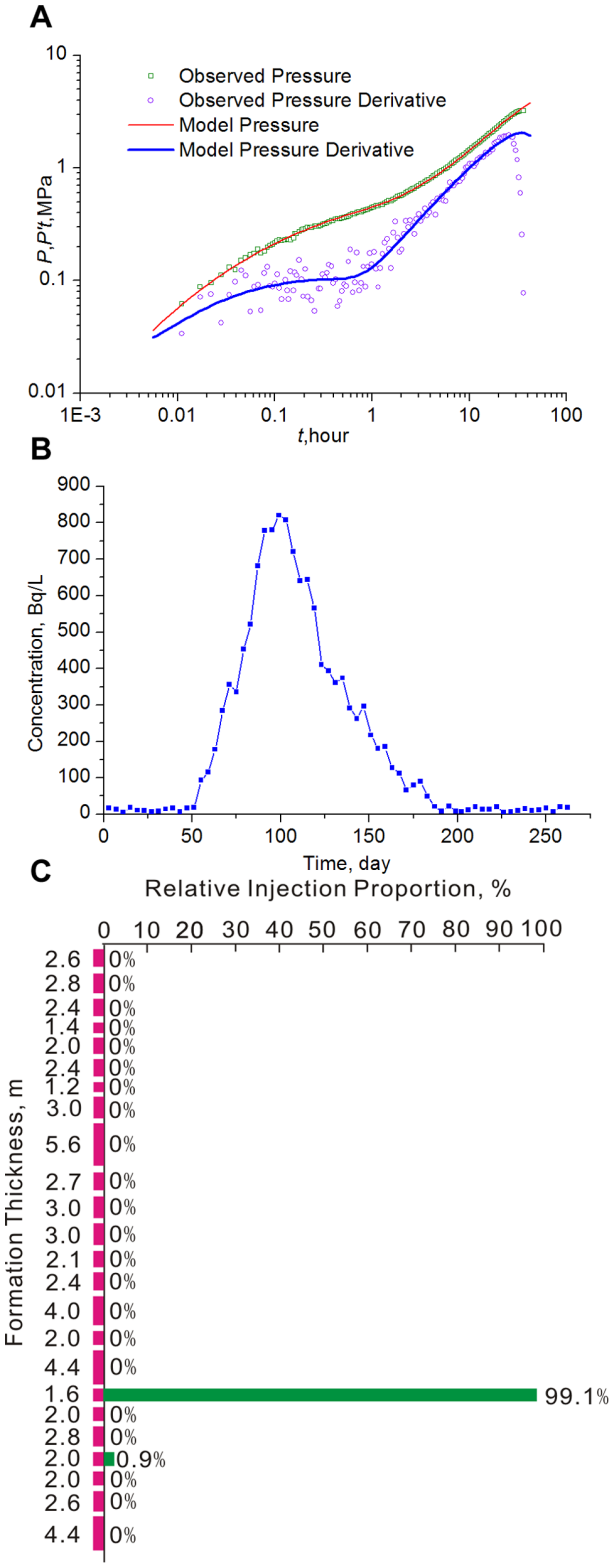
History match of the pressure transient test (A), interewell tracer concentration curve (B) and liquid production profile (C) for Well B1.

**Field Case 2:** Well B1 belongs to the Huabei Oilfield, located in the central area of Hebei Province in China. The formation and fluid parameters are shown in Table 6. Automatic history matching was also employed to fit the observed pressure and derivative with simulated data obtained from our model (Fig. 10A). Table 7 lists the estimated parameters of the preferential flow path, showing that although the permeability of the ordinary formation is only 0.01609μm^2^, the preferential flow path with permeability as high as 1.394 μm^2^ has evolved around this well. The interwell tracer test (Fig. 10B) and liquid production profile (Fig. 10C) also verify this conclusion. Considering the reservoir property, the tracer advance speed of 2.98 m/d is too high. Fig. 10C also reveals that although the net pay of this layer is 64.4 m, 99.1% of the production liquid should be attributed to the short segment with a thickness of only 1.6 m, which is very close to the estimated diameter of the preferential flow path (1.5136 m).

**Table 6:** Formation and fluid parameters for Well B1.

Parameter Name	Symbol	Value	Unit
Flow rate	*q*	30	m^3^/d
Fluid density	*ρ*	798	kg/m^3^
Fluid viscosity	*μ*	0.44	mPa·s
Reservoir porosity	*Ф*	0.23	dimensionless
Reservoir permeability	*k*	0.01609	μm^2^
Formation thickness	*h*	64.4	m
Compressibility	*C_t_*	0.001173	1/MPa

**Table 7:** Parameters of the preferential flow path for Well B1.

Parameter Name	Symbol	Value	Unit
Length of the preferential flow path	*L*	56.16	m
Permeability of the preferential flow path	*k_f_*	1.394	μm^2^
Vertical elevation of the preferential flow path	*z_p_*	16.35	m
Equivalent radius of the preferential flow path	*r_p_*	0.7568	m
Non-Darcy flow coefficient	*β*	2.51×10^9^	m^−1^
Storage constant	*C*	0.0703	m^3^/MPa

### Conclusions

Using the revised Forchheimer number recommended by Zeng and Grigg [23], the fluid flow behavior within a preferential path was examined. The results indicate that non-Darcy flow through a preferential path is possible for an actual oilfield and should be taken into account for its accurate characterization. This work establishes a hybrid analytical/numerical model for analyzing the pressure transient response of the preferential path with non-Darcy flow. The characteristics of the pressure transient behavior have also been discussed, as well as the influence of each corresponding parameter. Based on this model and the Gravitational Search Algorithm, an automatic matching technology has been supplied to estimate parameters of the preferential path with non-Darcy flow using the pressure transient test. Oilfield applications demonstrate that the characterization is in good agreement with interwell surveillance and dynamic analysis. Two examples from different oilfields are also provided to substantiate the validity of this approach. However, to characterize the preferential flow path more accurately and effectively, integration analysis with geological information, production logging test and several other methods is also essential and recommended.
